# Bacterial Sepsis: Challenges of Diagnosis and Treatment in a Teaching Hospital Southwest of Iran

**DOI:** 10.5812/jjm.9082

**Published:** 2014-03-01

**Authors:** Seyed Mohammad Alavi, Mehrdad Sharifi, Mehdi Eghtesad

**Affiliations:** 1 Infectious and Tropical Diseases Research Center, Jundishapur University of Medical Sciences, Ahvaz, IR Iran; 2 Ahvaz Health Center, Jundishapur University of Medical Sciences, Ahvaz, IR Iran; 3 Khuzestan Health Center, Jundishapur University of Medical Sciences, Ahvaz, IR Iran

**Keywords:** Bacterial Sepsis, Diagnosis, Therapeutics

## Abstract

**Background:**

Management of bacterial sepsis as a common cause of hospitalization and a life threatening clinical syndrome is a challenge. In previous studies, incorrect diagnosis of sepsis and unnecessary treatment have been frequently reported.

**Objectives:**

The aim of this study was to evaluate the diagnosis and treatment of cases with a primary diagnosis of sepsis.

**Patients and Methods:**

Of 410 medical files of patients with primary diagnosis of bacterial sepsis, 187 fulfilled our criteria and were enrolled in the study. The study was conducted in Razi Hospital of Ahvaz, southwest Iran, from 2009 to 2011. Data included demographic characteristics, underlying disease, clinical symptoms, laboratory and imaging findings, administrated antibacterial drugs, and nurses and doctors-analyzed notes. For evaluation of the diagnosis, patients were divided to two groups, sepsis group and pseudosepsis group, and for evaluation of the treatment, patients were categorized in appropriate and inappropriate treatment groups and compared using SSPS software version 16 by chi-square and fisher exact tests. P-values less than 0.05 were considered significant.

**Results:**

Out of 187 cases, 61 were in the intensive care unit (ICU), 98 in the infectious disease ward, and 28 in the internal medicine ward. Correct diagnosis of sepsis in the ICU, internal and infectious diseases wards were made in 16 (26.2%), 4 (14.3%) and 71 (72.4%) cases, respectively. Appropriate treatments for sepsis in the ICU, internal and infectious wards were applied in 12 (19.7%), 3 (10.7%) and 61 (78.2%) cases, respectively. Ninety-one patients (48.6%) were diagnosed correctly (true sepsis) and 76 (40.6%) were treated with proper regimes.

**Conclusions:**

Inappropriate and unnecessary use of antibiotics by patients with preliminary diagnosis of sepsis in our hospital, similar to other parts of the world, was high.

## 1. Background

According to the American Society of Critical Care Medicine, sepsis is a medical term defined as systemic inflammatory response syndrome (SIRS) in response to an infection. Infection can be suspected or proven if culture, stain, or polymerase chain reaction (PCR) tests for the specific pathogen are positive, or a clinical syndrome pathognomonic for the infection is present. Acceptable evidence for infection includes white blood cells (WBCs) in normally-sterile fluids (e.g. urine or cerebrospinal fluid (CSF)), evidence of a perforated viscous (by abdominal X-ray or CT scan), abnormal chest X-ray (CXR) consistent with pneumonia or petechial, purpura, or purpura fulminant (1). The therapy of sepsis depends on intravenous fluids, appropriate broad spectrum antibiotics (2), surgical drainage of infected fluid collections, and appropriate support for organ dysfunction (2, 3). 

Sepsis as a clinical syndrome is called to symptomatic bacteremia with or without organ impairment. This medical term is often used in patients admitted with fever and leukocytosis (4). The commonest infectious cause of sepsis in hospitals include severe community-acquired and nosocomial pneumonias, pyelonephritis, intravenous line infections, septic pulmonary emboli, viral hepatitis, antibiotic-associated diarrhea/colitis, infected decubitus ulcers, and intra-abdominal or pelvic infection due to perforation, trauma, or surgery. In most cases, causes other than infections but with these two signs are also included. In fact, over-diagnosis occurs in many cases of pseudosepsis (5). Sepsis is one of the major causes of death in the world, particularly in developing and undeveloped countries (6, 7). 

Prognosis of sepsis depends on several factors such as the underlying diseases, immune status, and early appropriate empirical treatment with effective antibiotics (8). Distinguishing true sepsis from pseudosepsis is important for immediate initiation of empiric treatments. Pseudosepsis is one of the causes of unnecessary antibiotic usage and wrong diagnosis of the disease can cause bacterial resistance. The basic criteria for diagnosis of sepsis include at least two of the four signs of SIRS (abnormal body temperature, heart rate, respiratory rate, and WBC count) in the presence of infection (9, 10). 

Patients with sepsis are often admitted to hospitals for immediate treatment with intravenous fluid and broad spectrum antibiotics based on the source of infection (1). In communities where the indiscriminate use of antibiotics is common, diagnostic difficulties in dealing with very ill patients may lead to inappropriate treatment. Unnecessary or inappropriate use of antimicrobials in addition to drug toxicity, increased morbidity and health care costs, may cause the emergence of antimicrobial resistance. Antimicrobial use has been reported to be incorrect or not indicated in 9 - 64% of inpatients (11).

According to the published reports, Iran is among countries with high antimicrobial resistance (12-15). According to the available evidences, hospitals are places where unnecessary antibiotics are frequently prescribed (12, 13, 15). To the best of our knowledge and from more than 25 years of experience in hospitals, bacterial sepsis is a common hospital disease in which antibiotics are prescribed. Routinely, antibiotics in combination (two or more) are prescribed for patients hospitalized with a primary diagnosis of sepsis, a significant number of whom are in fact falsely diagnosed and antibiotics are incorrectly prescribed for them. Even when the diagnosis of sepsis is correct, the medication is not administered properly; for instance, inappropriate combination of antibiotics, incorrect dose, or inadequate duration of treatment. 

To solve the problem of antibiotics overuse in the hospital, firstly, the situation of diagnosis and treatment of the disease should be identified, and next, problems, bottlenecks, and high-risk areas and departments should be determined. Field studies to identify factors affecting the unnecessary use of antimicrobials are the most urgent measures for controlling this problem. Seemingly, such study has not been conducted even in a single region.

## 2. Objectives

The aim of this study was to review cases with primary diagnosis of sepsis and determine incorrect diagnosed cases of sepsis as well as unnecessary consumptions of antibiotics in a teaching hospital.

## 3. Patients and Methods

### 3.1. Study Design and Population

In a retrospective study, medical files of 410 admitted patients with a primary diagnosis of bacterial sepsis in Razi Hospital affiliated to Jundishapur University of Medical Sciences in Ahvaz, southwest Iran, from 2009 to 2011 were studied. The study was approved by the Research Council of Infectious Disease department. The inclusion criteria were age of over 18 and admission due to sepsis. The exclusion criteria were incomplete records, leaving the hospital for any reason, and HIV-associated infections.

### 3.2. Methods

Sepsis-related data were extracted from patients’ medical files, including demographic characteristics, underlying disease, clinical symptoms, laboratory and imaging findings, administrated antibacterial drugs, and doctors and nurses’ notes, and analyzed. We used bacterial sepsis guideline and its criteria to differentiate patients who have just been diagnosed (true sepsis) from those with a false diagnosis (pseudosepsis), as well as differentiate properly treated patients from inappropriately treated ones. Patients were considered correctly diagnosed with sepsis if the diagnosis was based on two of the four SIRS criteria shown in Table 1 in the presence of bacterial infection evidence (culture, Gram staining, antigen detection tests, serology tests, imaging and clinical findings suggesting of infection).

Patients with SIRS criteria and without bacterial infection evidence were classified as pseudosepsis. Patients were defined as appropriately treated if they received antibiotic regimen with full coverage against most probable pathogens, regarding the age of patient and site of infection (e.g. empirical treatment was recommended for sepsis due to urinary duct, pulmonary or soft tissue origin), or based on the bacterial culture and antibiogram results (1). Both patients with pseudosepsis (unnecessary treated) and incorrect antibiotic regimen, improper dose or duration, were defined as inappropriately treated. For evaluation of the diagnosis, patients were divided to two groups: sepsis (SG) and pseudosepsis (PSG), and for evaluation of the treatment, patients were divided to two groups: appropriate (AT) and inappropriate (IAT) treatments.

**Table 1.** Systemic Inflammatory Response Syndrome

Finding	Value
**Temperature**	< 36 °C (96.8 °F) or > 38 °C (100.4 °F)
**Heart rate**	> 90/min
**Respiratory rate**	> 20/min or PaCO_2 _< 32 mmHg (4.3 kPa)
**WBC ** ^**a**^	< 4 × 10 ^9^/L ( < 4000/mm ³), > 12 × 10 ^9^/L (>12,000/mm ³), or 10 % bands

^a^ Abbreviations: WBC, white blood cell

### 3.3. Statistical Analysis

Data from different groups were compared using SSPS-16 by chi-square and fisher exact test; P values less than 0.05 were considered as significant. 

## 4. Results

Out of 410 patients, 223 were excluded according to the exclusion criteria. Of the remaining 187 cases, 61 were in the intensive care unit (ICU), 98 in the infectious disease ward, and 28 in the internal medicine ward. The number of patients correctly diagnosed with sepsis in the ICU, internal and infectious wards were 16 (26.2%), 4 (14.3%) and 71 (72.4%), respectively (Table 2). Appropriate treatment of sepsis in the ICU, internal and infectious wards were applied for 12 (19.7%), 3 (10.7%) and 61 (78.2%) patients, respectively (Table 3). 

Ninety-one patients (48.6%) were diagnosed correctly (true sepsis) and 76 (40.6%) treated with proper regimens. In 15 of 91 patients with true sepsis, medications were not administered properly; inappropriate combination of antibiotics (nine patients), incorrect dose (four) and inadequate duration of treatment (two). As shown in Tables 2 and 3, the infectious disease ward had the highest correct diagnosis and treatment rates (78% and 80.3%, respectively) and to the internal medicine ward had the lowest (4.4% and 3.9%, respectively). Both true sepsis and appropriate treatment in the infectious diseases ward were significantly higher than other wards (P = 0.000).

Blood culture of 31, urine culture of 13, and Gram staining of respiratory secretion of 19 patients had positive results. From the total of 91 individuals with true sepsis, only 35 (38.5%) were diagnosed based on the isolation of microorganisms. The most common isolated microorganisms were: *Staphylococcus* *aureus* (16/35, 45.7%), *Escherichia coli* (10/35, 28.5%), *Klebsiella pneumonia* (4/35, 11.4%), *Pseudomonas aeruginosa* (8/35, 22.8%), and coagulase-negative* staphylococci* (*S. epidermidis* and *S. haemolyticus*) (6/11, 17.1%). More than one microorganism was isolated from approximately 30% of patients. More than 75% of K*lebsiella* and *Pseudomonas* and about 87% of methicillin-resistant *S. aureus* (MRSA) were isolated from the ICU patients. Bacterial resistance to at least one common antibiotic such as Ampicillin, Cephalothin, Cotrimoxazole, Aminoglycoside and Ceftriaxone was observed in 90% of cases.

**Table 2.** Comparison of Patients With True Sepsis and Pseudosepsis in Different Hospital Departments

Department	SG ^a^, No. (%)	PSG ^a^, No. (%)	P Value
**ICU ** ^**a**^	16 (17.6)	45 (46.9)	< 0.001
**Internal medicine**	4 (4.4)	24 (25)	< 0.001
**Infectious diseases ** ^**b**^	71 (78)	27 (28.1)	0.000
**Total**	91 (100)	96 (100)	

^a^ Abbreviations: SG; sepsis group, PSG; pseudosepsis group, ICU; intensive care unit.

^b^ statistically significant.

**Table 3.** Comparison of Appropriately and Inappropriately Treated Patients in Different Hospital Departments

Department	ATG ^a^, No. (%)	IATG ^a^, No. (%)	P Value
**ICU ** ^**a**^	12 (15.8)	49(44.1)	< 0.001
**Internal medicine**	3 (3.9)	25 (22.5)	< 0.001
**Infectious diseases ** ^**b**^	61 (80.3)	37 (33.4)	0.000
**Total**	76 (100)	111 (100)	

^a^ Abbreviations: ATG; appropriate treatment group, IATG; inappropriate treatment group, ICU; intensive care unit.

^b^ Statistically significant.

## 5. Discussion

Widespread and unnecessary use of antibiotics is a major cause of microbial resistance. Incorrect and over-diagnosis of infectious diseases are the main reasons for excessive prescription of antibiotics. The present study showed that more than half of the patients hospitalized due to primary diagnosis of sepsis, actually had no sepsis. We found that diagnosis of sepsis in the ICU as other parts of the hospital is often inaccurate, while in the infectious diseases ward, sepsis diagnosis is mostly correct (16/61, 26.2% vs.71/98, 72.5%). Interpretation of this situation is simply not possible, but it can be suggested that management of sepsis in the ICU and internal wards is usually performed by anesthesiologists and internists who are unfamiliar with infectious diseases, whereas in the infectious diseases ward, infectious diseases experts with specialties in this field use their skills in applying sepsis guidelines for diagnosis. Due to lack of similar studies, comparing these finding with other studies is impossible.

In this study, we also found that approximately 60% of admitted patients had been under unnecessary treatments. Similar reports of sepsis over-diagnosis and unnecessary antibiotic usage in hospitalized patients with primary diagnosis of sepsis are available (5, 11, 16-19). Treatment of sepsis in the ICU as in the internal ward was inappropriate, whereas in the infectious diseases ward, the treatment was mostly appropriate (12/61, 19.7% vs. 61/98, 62.2%). The reason for this difference is that infectious diseases specialists, according their knowledge, prescribes a combination of antibiotics against the most common microorganisms causing bacterial sepsis empirically, considering the source of infection or based on culture and antibiogram results. Due to the differences in design, studied population, method of sampling and age groups between our study and the abovementioned reports, comparison of our findings with other studies is biased and impossible.

In our study, the diagnosis was based on the isolation of microorganisms in only 38.5% of cases. In fact, more than 60% of cases underwent empirical treatment based on the physician’s decision. It seems that technical deficiencies in the laboratories might be among important causes of unnecessary and over-prescription of antibiotics in the region under study as well as other developing countries (9).

In this study, similar to the previous studies, the most common isolated microorganisms were: *S. aureus*, *E. coli*, *K. pneumonia*, *P. aeruginosa* and coagulase-negative* staphylococci* *(S. epidermidis* and *S. haemolyticus*). This finding is in agreement with most published studies (16, 17, 20). considering this issue when dealing with sepsis may help in administration of proper antibiotics and prevention of treatment failure. Microbial resistance to at least one drug was observed in 90% of our cases. The microbial resistance rates were higher than the rates reported by the previous studies (9, 18, 21). These findings indicate high levels of microbial resistance in our country; an issue repeatedly warned by the previous studies (19, 22).

### 5.1. Strengths and Limitations of the Study

To the best of our knowledge and from researches in the most famous scientific websites, this study is unique and no similar study was found. This study was retrospective; therefore, access to the target data was difficult and with low reliability. However, this study might be useful as a preliminary for further prospective studies.

### 5.2. Conclusions

Inappropriate and unnecessary use of antibiotics in patients with preliminary diagnosis of sepsis in our hospital outside the infectious disease ward was higher than other parts of the world, which was because of the lack of enough skilled physicians in bacterial sepsis management.
